# A novel nudivirus infecting the invasive demon shrimp *Dikerogammarus haemobaphes* (Amphipoda)

**DOI:** 10.1038/s41598-020-71776-3

**Published:** 2020-09-09

**Authors:** Thomas W. Allain, Grant D. Stentiford, David Bass, Donald C. Behringer, Jamie Bojko

**Affiliations:** 1 School of Forest Resource and Conservation, University of Florida, Gainesville, FL 32611 USA; 2 International Centre of Excellence for Aquatic Animal Health, Centre for Environment, Fisheries and Aquatic Science (Cefas), Weymouth, Dorset, DT4 8UB UK; 3 Centre for Sustainable Aquaculture Futures, Biosciences, University of Exeter, Stocker Road, Exeter, EX4 4PY UK; 4 Fisheries and Aquatic Sciences, University of Florida, Gainesville, FL 32653 USA; 5 Emerging Pathogens Institute, University of Florida, Gainesville, FL 32611 USA; 6 School of Health and Life Science, Teesside University, Middlesbrough, TS1 3BA UK; 7 National Horizons Centre of Excellence in Bioscience Industry, Teesside University, Darlington, DL1 1HG UK

**Keywords:** Ecology, Virology

## Abstract

The *Nudiviridae* are a family of large double-stranded DNA viruses that infects the cells of the gut in invertebrates, including insects and crustaceans. The phylogenetic range of the family has recently been enhanced via the description of viruses infecting penaeid shrimp, crangonid shrimp, homarid lobsters and portunid crabs. Here we extend this by presenting the genome of another nudivirus infecting the amphipod *Dikerogammarus haemobaphes*. The virus, which infects cells of the host hepatopancreas, has a circular genome of 119,754 bp in length, and encodes a predicted 106 open reading frames. This novel virus encodes all the conserved nudiviral genes (sharing 57 gene homologues with other crustacean-infecting nudiviruses) but appears to lack the p6.9 gene. Phylogenetic analysis revealed that this virus branches before the other crustacean-infecting nudiviruses and shares low levels of gene/protein similarity to the *Gammanudivirus* genus. Comparison of gene synteny from known crustacean-infecting nudiviruses reveals conservation between *Homarus gammarus nudivirus* and *Penaeus monodon nudivirus*; however, three genomic rearrangements in this novel amphipod virus appear to break the gene synteny between this and the ones infecting lobsters and penaeid shrimp. We explore the evolutionary history and systematics of this novel virus, suggesting that it be included in the novel *Epsilonnudivirus* genus (*Nudiviridae*).

## Introduction

The family *Nudiviridae* comprises a group of non-occluded, double-stranded DNA (dsDNA) viruses infecting arthropods. The family includes two recognised genera, *Alphanudivirus* and *Betanudivirus*, with two other genera, *Gammanudivirus* and *Deltanudivirus*, recently proposed to contain aquatic-host-infecting viruses ^1,2^. *Alphanudivirus* contains two species; *Gryllus bimaculatus nudivirus* and *Oryctes rhinoceros nudivirus*, while *Betanudivirus* contains a single species, *Heliothis zea nudiviru*s ^3^. In addition, several other insect-infecting nudiviruses have been morphologically and/or genomically characterized but remain to be formally recognised ^4^. In recent years, closely related viruses have been identified infecting marine crustaceans, using genomics and ultrastructural data. The most complete descriptions include *Penaeus monodon nudivirus* (PmNV) infecting the farmed penaeid shrimp *Penaeus monodon*^1^ and recently, *Homarus gammarus nudivirus* (HgNV) infecting juveniles of the European lobster, *Homarus gammarus*^2^.

Other crustaceans are also infected with putative nudiviruses, often referred to in the published literature as “bacilliform viruses” in lieu of available genomic data^5–11^. In addition to descriptions of putative nudiviruses in these decapod crustaceans, observations of nudivirus-like infections of Amphipoda have also been reported; e.g. for *Dikerogammarus villosus* ^12^, *Dikerogammarus haemobaphes* ^11^, *Gammarus roeselii* ^8^, *Pontogammarus robustoides* ^7^ and *Gammarus varsoviensis* ^7^. In all cases, putative nudivirus infection is observed within cells of the hepatopancreas, causing nuclear hypertrophy but no observable host immune response to infection, in histological Section^8,11^. Histopathologically, nudiviral infection results in nuclear hypertrophy of the hepatopancreatocytes, caused primarily by a growing viroplasm; this is explored in full for the virus in *D. haemobaphes* in Bojko et al. ^11^. Also in *D. haemobaphes*, infection prevalence of up to 77.7% has been correlated with altered behaviour in infected animals ^11^. The behavioural change was associated with increased activity, which positively correlated with viral burden, potentially indicating some benefit to viral transmission through increased movement ^11^. Many of the amphipod hosts in which putative nudiviruses have been reported are non-native or invasive species present outside of their native ranges. Infection with this viral family may therefore have potential for transboundary transmission when their hosts are present in their invasive range ^7,10,11^.

Genomic data is currently lacking for all the putative nudiviruses infecting amphipods. In this study, we provide full genome characterisation of a nudivirus infecting the amphipod *D. haemobaphes* collected from outside of its native range. We use these data to provisionally name the virus as *Dikerogammarus haemobaphes nudivirus* (DhNV) and place the virus within a newly suggested genus *Epsilonnudivirus* of the family *Nudiviridae*.

## Results

### Genome structure of DhNV

**Figure 1:**
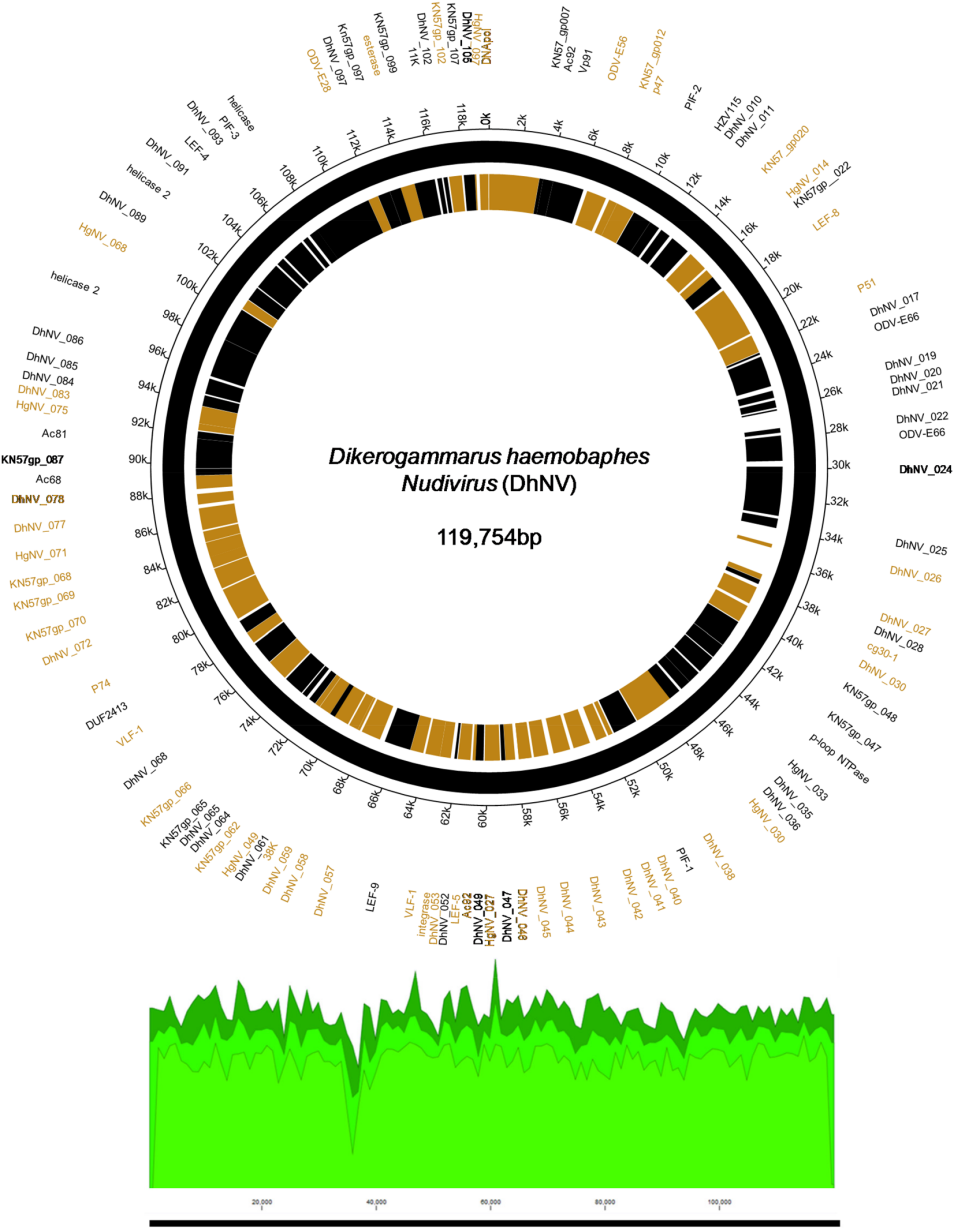
The ‘*Dikerogammarus haemobaphes nudivirus*’ circular genome with open reading frames (ORF) graphically plotted across the 119,754 bp sequence. Locus tags for each similar virus are denoted in the place of DhNV genes, where possible. Gold regions and text indicate predicted genes on the positive strand and black regions and text indicate those on the negative strand. Below the genome, the green plot displays genome coverage across the genome using the MiSeq and HiSeq trimmed sequence reads*.* ORFs with significant similarity (e-value < 0.001) to other nudivirus genes are listed as the reference gene number on the corresponding viral genome. The circular plot was developed in Circa (www.omgenomics.com/circa/) and the coverage map from CLC genomics workbench v.12 (Qiagen).

The circular genome of DhNV is 119,754 bp and contains 106 hypothetical ORFs, with 56 on the positive strand and 50 on the negative strand (Fig. 1). Fifty-nine of the ORFs met our comparative e-value threshold of < 0.001 and were directly comparable to other members of the *Nudiviridae*. Up to 17% and 37% of the ORFs aligned most closely with genes from HgNV and PmNV, respectively. Two ORFs, DhNV_008 and DhNV_002, scored above 50% similarity to protein sequences on BLASTp. DhNV_008 was 52.33% similar to *pif-2* from HgNV (QBB28614) with *per os* infectivity as the only identified protein domain. DhNV-002 was 50% similar to a hypothetical protein from PmNV (YP_009051845) where the cytoplasmic, non-cytoplasmic, tmhelix, and transmembrane domains were identified. Among the 47 ORFs that provided no similarity to other protein sequences within the threshold, InterProScan assessment identified 20 ORFs with functional domains. A protein signature match to the inhibitor of apoptosis repeat superfamily in DhNV_059 may indicate the presence of a homolog of the *Iap* nudivirus gene; however, BLASTp annotations did not yield any similarity results to the *Iap* gene found in other nudiviruses. The remaining 19 ORFs contained proteins with Zinc finger domains (DhNV_045), Tmhelix (DhNV_044), signal peptide (DhNV_084), p-loop containing nucleoside triphosphate hydrolase (DhNV_047), non-cytoplasmic domain (DhNV_025, 028, and 057), disorder predictions (DhNV_011, 020, 024, 072, 077, and 078), predicted to be cytoplasmic (DhNV_019 and 065), or had ‘coil’ feature(s) (DhNV_022, 026, 042, and 086).

Seven genes involved with DNA processing were identified: *DNA polymerase, helicase,* two copies of *helicase 2, integrase, DNA ligase,* and *fen-1*. Five genes involved with RNA transcription were also identified: *p47, lef-4, lef-5, lef-8,* and *lef-9.* Eight genes known to be involved in *per os* infectivity were found: *vp91* (*pif-8*)*, pif-1, pif-2, pif-3, odv-e28* (*pif-4*)*, odv-e56* (*pif-5*)*, ac68* (*pif-6*)*,* and *p74* (*pif-0*), along with the *11 k* gene. Nine genes can be grouped into packaging, assembly, and release: two copies of *ac92* (*p33*)*,* two copies of *odv-e66,* two copies of *vlf-1*, *38 k, ac81, and 31 K* (*vp39*). The recently identified *p6.9* gene in crustacean-infecting nudivirus genomes, a baculovirus core gene associated with encapsulation of the viral genome, could not be found in DhNV despite similarity searches and checks for hypothetical SRSR repeat regions common to the p6.9 protein. A putative DUTPase, cg30-1, p-loop NTPase, guanosine monophosphate kinase, esterase, and *p51* were also identified. Definitively, 20 out of the 21 core baculovirus genes conserved among nudivirus were identified in the DhNV genome (Table 1).

Multiple genes were specific to DhNV; however, some were similar to other protein groups from various taxa. One ORF, DhNV_076, revealed 31.98% to 35.97% similarity to proteins from seven different organisms. The LOC108666550-like protein from HgNV is 32.35% similar (85% coverage) to DhNV_076 (Table 1). Uncharacterized proteins from *Hyalella azteca* (QBB28676, sim. 35.97% cov. 82%), an amphipod native to North America; *Penaeus vannamei* (XP_027235023, sim. 34.07% cov. 83%), the Whiteleg shrimp; *Crassostrea gigas* (XP_011433877, sim. 31.98% cov. 85%)*,* the Pacific Oyster; *Armadillidium vulgare* (RXG54766, sim. 34.15% cov. 83%), the common pill-bug; and *Tigriopus californicus* (TRY76277, similarity 33.26% coverage 88%), a North American coastal copepod, all met the e-value threshold, as did the actin-binding IPP-like protein from *Brachionus plicatilis* (RMZ96256 similarity 33.65% coverage 87%). Only the undescribed PANTHER protein family, PTHR38566, was identified as a domain in DhNV_076.

### Gene synteny among the *Epsilonnudivirus*, *Gammanudivirus *and *Deltanudivirus* genera

**Figure 2:**
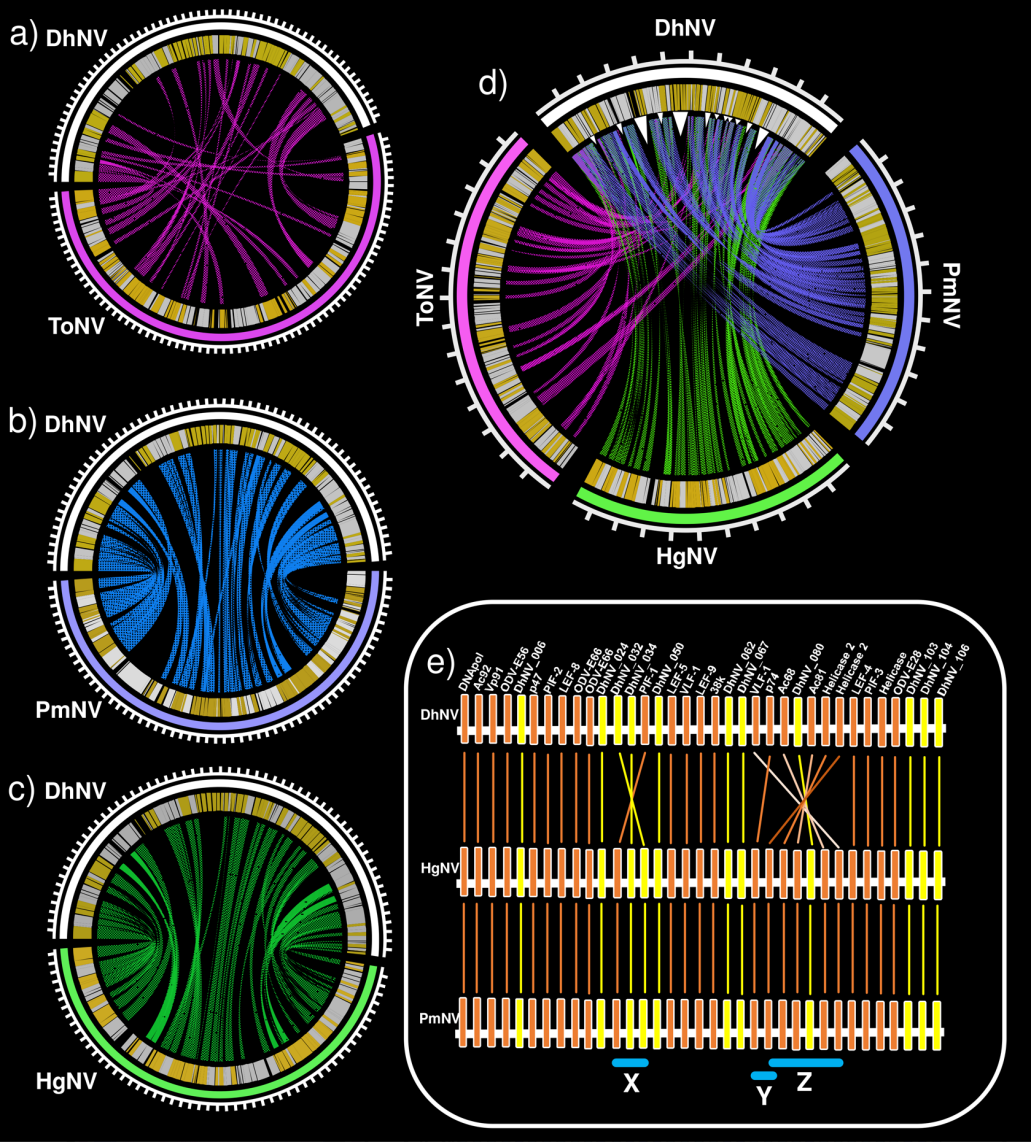
(**a**) Gene synteny among the *Epsilonnudivirus*, *Gammandivirus* and *Deltanuvirus* reveals a gene order among decapod-infecting nudiviruses, which is missing from peracarid-infecting nudiviruses. The genomes are annotated with positive strand (gold) and negative strand (silver) coding regions. In Fig. 2a, *Dikerogammarus haemobaphes nudivirus* gene synteny (white) is compared to Tipula oleracea nudivirus gene synteny (pink). Ribbons connecting the two genomes link up the homologous gene and its location on the viral genome. Scale ticks = 2 kb. The comparative plots were developed in Circa (www.omgenomics.com/circa/). (**b**) *Dikerogammarus haemobaphes nudivirus* gene synteny (white) is compared to *Penaeus monodon nudivirus* gene synteny (blue). Ribbons connecting the two genomes link up the homologous gene and its location on the viral genome. Scale ticks = 2 kb. The comparative plots were developed in Circa (www.omgenomics.com/circa/). (**c**) *Dikerogammarus haemobaphes nudivirus* gene synteny (white) is compared to *Hommarus gammarus nudivirus* gene synteny (green). Ribbons connecting the two genomes link up the homologous gene and its location on the viral genome. Scale ticks = 2 kb. The comparative plots were developed in Circa (www.omgenomics.com/circa/). (**d**) All four virus are compared together, indicating regions of novelty in the *Dikerogammarus haemoabphes nudivirus* genome. The white triangles on the DhNV genome highlight the areas of novel sequence information that do not correspond to genes on the other nudivirus genomes. Scale ticks = 10 kb. The comparative plots were developed in Circa (www.omgenomics.com/circa/). (**e**) The crustacean nuidviruses contain 24 nudivirus core genes (VLF-1, ODV-E66 and Helicase 2 are duplicated) (p6.9 is missing from *Dikerogammarus haemobaphes nudivirus*) (orange) and 11 other gene homologs conserved across the crustacean-infecting nudiviruses (yellow). These conserved homologues are based on similarity, synteny and functional identity. Comparison with DhNV (peracarid-infecting nudivirus) highlights three main areas of gene reorganization. ‘X’ corresponds to a rearrangement of the DhNV_032, DhNV_34 and *pif-1* genes, ‘Y’ corresponds to a rearrangement of the *vlf-1* and *p74* genes and finally, ‘Z’ corresponds to a rearrangement of six genes (*vlf-1*, *ac68*, DhNV_080, *ac81* and both *helicase 2* homologues). The *Gammanudivirus* members that infect decapods share the same gene synteny across these conserved motifs.

A comparison of gene synteny between DhNV and three other nudiviruses determined that DhNV had a different gene synteny to members of the *Gammanudivirus* and *Deltanudivirus* genera (Fig. 2). Comparison between ToNV and DhNV revealed a high level of genomic rearrangement, where the 32 genes that showed genetic similarity (e < 0.001) with those ORFs on the DhNV genome were located across the respective genomes, showing little conserved synteny (Fig. 2a). Comparison between DhNV and PmNV/HgNV revealed higher levels of gene synteny (Fig. 2b,c). A comparison using all three viruses identified 12 major regions of genetic novelty in the DhNV genome (Fig. 2d). This included 47 hypothetical ORFs that were unique to DhNV and showed little genetic/protein relatedness to other nudiviruses within the e-value threshold of < 0.001, one of which (DhNV_070) showed highest similarity to a gene from *Pyricularia oryzae*, a fungal plant pathogen, and another (DhNV_029) with highest similarity to *Sucra jujuba nucleopolyhedrovirus* (Table 1).

Using the protein similarity data, we determined that there were 11 crustacean-infecting nudivirus genes (DhNV_006, 024, 032, 034, 050, 062, 067, 080, 103, 104 and 106) that show conservation across the crustacean-infecting nudiviruses (Table 1) (i.e. present in PmNV, HgNV and DhNV) but appear absent from other nudiviruses that do not infect crustaceans. Using these genes in addition to the conserved baculovirus core genes across DhNV, HgNV and PmNV ^1,2^, 35 genes were comparable in a “gene-block” fashion relative to their genomic loci (Fig. 2e). This revealed three major rearrangement events. Reordering of the DhNV_032, DhNV_034, and *pif-1* gene block, which is reversed in PmNV and HgNV (Fig. 2, ‘X’). Reordering of the *vlf-1* and *p74* gene block, which is reversed in PmNV and HgNV (Fig. 2, ‘Y’) and a larger rearrangement of 6 genes (*vlf-1, ac68,* DhNV_080, *ac81,* and both copies of *helicase 2*), which is reversed in PmNV and HgNV and overlaps the ‘Y’ rearrangement event (Fig. 2, ‘Z’).

### Morphological and phylogenetic comparison to other *Nudiviridae*

**Figure 3:**
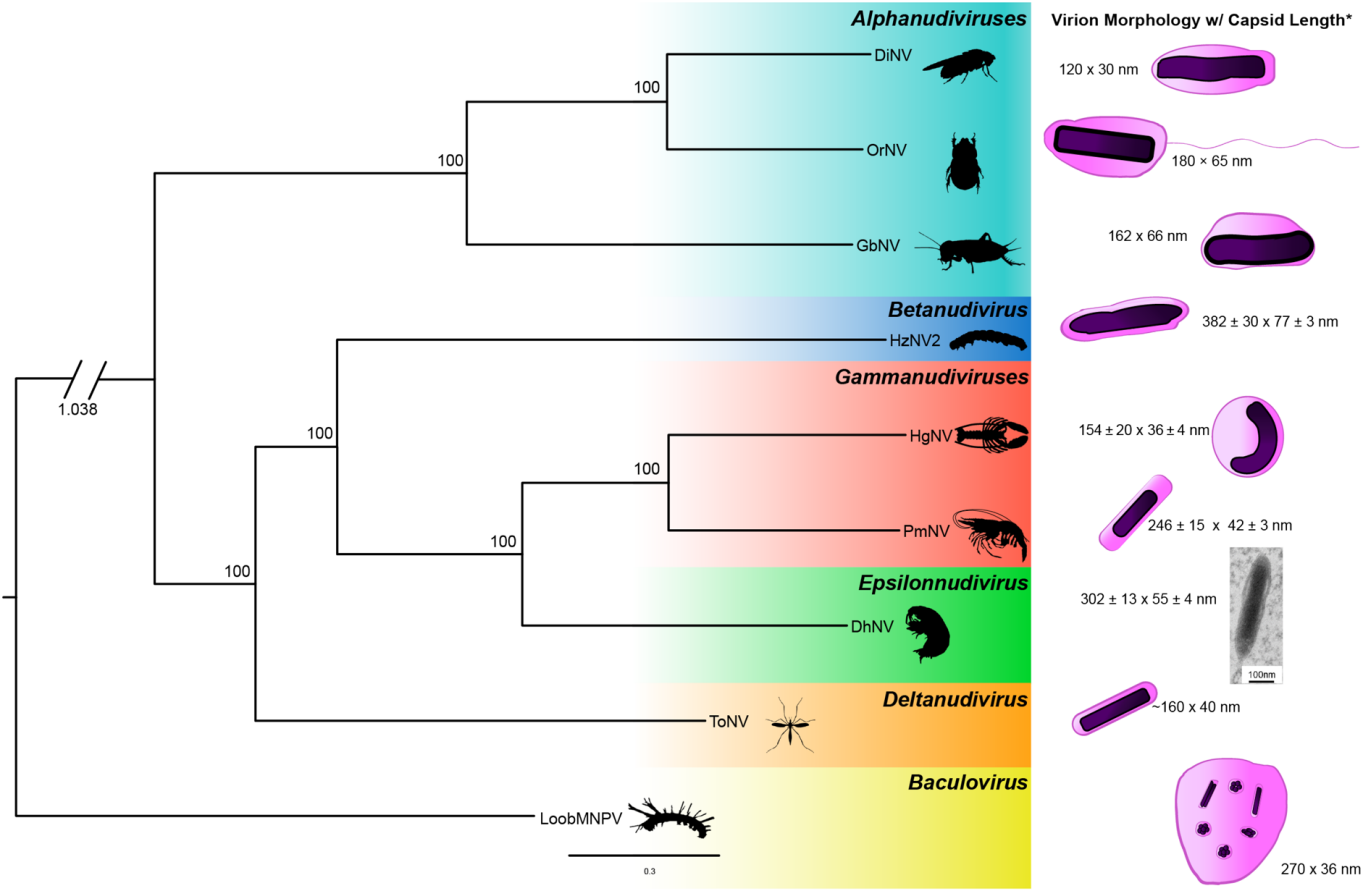
A concatenated phylogeny using 18 core nudivirus genes among 8 nudiviruses with LoobMNV as an outgroup. Node labels indicate bootstrap support in percent. Nudivirus genera are displayed in coloured groups. Illustrations of virion morphology are presented to the right of the tree and are based off electron micrographs of electron dense cores surrounded by a membrane. Capsid length approximations and averages are displayed from relevant publications cited in the methods. Genome accession numbers include: DiNV (NC_040699), OrNV (MN623374), GbNV (NC_009240), HzNV2 (NC_004156), HgNV (MK439999), PmNV (NC_024692), DhNV (MT488302), ToNV (NC_026242) and LoobMNPV (NC_043520). The tree was annotated in FigTree v.1.4.3. For additional information on DhNV virion morphology and pathology, please consult Bojko et al.^11^.

A concatenated maximum likelihood phylogenetic analysis of eight nudiviruses and one baculovirus (outgroup) using 18 core nudivirus genes (see Sect. 4) supported the positioning of DhNV outside of the two crustacean-infecting nudiviruses with bootstrap values of 100% (Fig. 3). Within this grouping, DhNV is an early branching member of the *Gammanudivirus* genus and may constitute a different genus altogether. The *Betanudivirus* genus branches outside of the *Gammanudivirus* cluster and the *Deltanudivirus* member ToNV is the earliest branching member of these three genera. The *Alphanudivirus* group represents the most phylogenetically distinct nudivirus genus represented on our diagram (Fig. 3).

Illustrations of virus morphology provide another dimension of comparison among the *Nudiviridae* species (Fig. 3). DhNV virions consist of a double membrane surrounding an electron-dense core measuring (n = 30, mean ± SD) 302 ± 13 nm in length and 55 ± 4 nm at its diameter ^11^. The rod-shaped structure is maintained across all the nudiviruses. DhNV represents one of the larger nudiviruses discovered to date, second to HzNV2, which has a length of 382 ± 30 nm.

**Figure 4:**
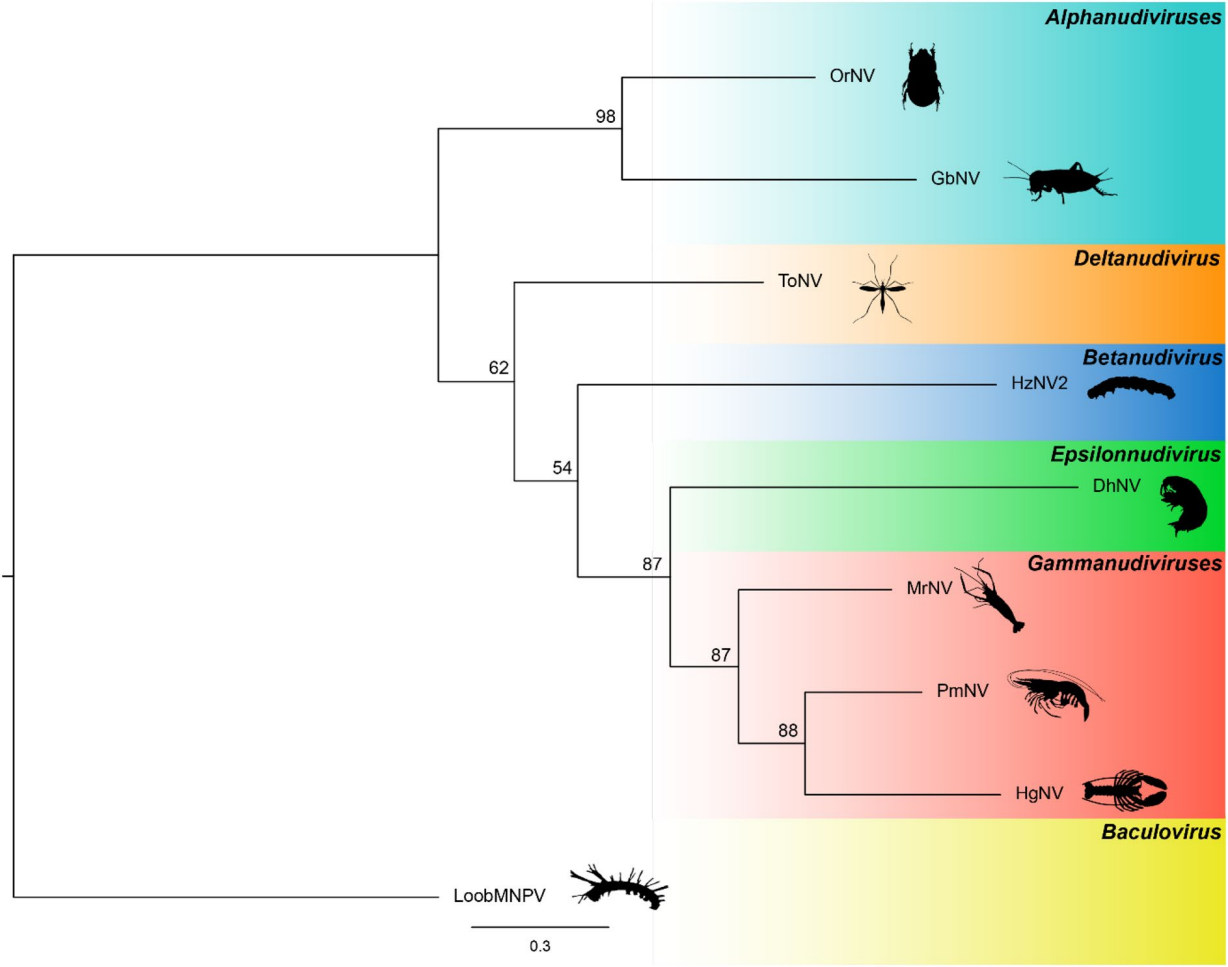
A concatenated phylogeny using *iap* and *pif-2* genes from 8 nudiviruses with LoobMNPV as an outgroup. Nodes are assigned bootstrap support values from 1,000 bootstrap replicates. Accession numbers include: OrNV (MN623374), GbNV (NC_009240), ToNV (NC_026242), HzNV2 (NC_004156), DhNV (MT488302), MrNV (JQ804994; JQ804993), PmNV (NC_024692), HgNV (MK439999) and LoobMNPV (NC_043520). The tree was annotated in FigTree v.1.4.3.

A second concatenated maximum likelihood phylogenetic analysis of putative *iap* and *pif-2* genes supported DhNV as an earlier branch of the crustacean-infecting nudiviruses. The addition of *Macrobrachium rosenbergii nudivirus* CN-SL2011 (MrNV) (NCBI:txid1217568), which only has the aforementioned genes available, branched in the *Gammanudivirus* genus. ToNV (*Deltanudivirus*) is the earliest branch of these genera, followed by HzNV2 (*Betanudivirus*), and the four crustacean-infecting nudiviruses. The *Alphanudiviruses* represent the most phylogenetically distinct lineage among nudiviruses in this tree (Fig. 4), following the same general phylogenetic theme as the details in Fig. 3.

## Discussion

We provide a full genome characterisation of DhNV, a novel member of the *Nudiviridae* infecting the freshwater amphipod host, *Dikerogammarus haemobaphes*. The genome size, ORFs and morphology of this virus correspond with related viruses from crustaceans and insects. The identification of this virus is discussed relative to its genetic and protein content, its gene synteny and the gene synteny of related viruses, and finally, its phylogenetic relatedness to other *Nudiviridae*. These combined data suggest a novel genus may be appropriate: *Epsilonnudivirus*.

### A novel member of the *Nudiviridae* (*Epsilonnudivirus*) from an amphipod

Using a combination of the core genes conserved across the *Nudiviridae*, we show that DhNV is most related to the *Gammanudivirus* genus; however, with a low level of protein similarity at most loci (< 50%) it seems pertinent to explore the erection of a new genus. Our concatenated phylogenetic analysis of eight nudiviruses representing four genera is concordant with previously published trees ^2^. DhNV appears to branch early from the three marine nudiviruses (Figs. 3, 4), suggesting an ancestral position to the HgNV, MrNV and PmNV.

The DhNV genome encoded all core nudivirus genes, apart from *p6.9*, a nucleotide-binding protein. These proteins function for DNA processing, RNA transcription, and *per os* infectivity ^13^. The *p6.9* gene, which is responsible for the encapsulation of the viral genome, is characterized by a serine-arginine repeat region that could not be identified from the DhNV genome and was not present at the predicted locus where *p6.9* lies in other *Gammanudivirus* members: between *lef-5* (DhNV_051) and *vlf-1* (DhNV_055). In addition to the core genes (n = 24, including three repeat homologues), most genes show similarity to other *Nudiviridae* under a conservative e-value threshold (< 0.001), providing strong evidence that this virus belongs within the *Nudiviridae* (Table 1).

The primary source of protein similarity information for DhNV ORF’s came from PmNV and HgNV, two genomically characterised viruses from the *Gammanudivirus* genus. *Gammanudivirus* members contain unique apoptosis inhibitor genes that lack a predicted RING domain ^1^ and appear twice in the HgNV genome. DhNV_059 represents a homolog of the *Iap* nudivirus gene in DhNV, where an inhibitor of apoptosis repeat domain was detected but is relatively different from existing Baculovirus homologues ^1^. In addition to family-level gene conservation, we identified 11 “crustacean-infecting nudivirus” genes that are conserved among those that infect crustaceans. Using a gene-block approach, we identified that PmNV and HgNV share gene synteny, where the DhNV genome exhibits three reorganization events, termed ‘X’, ‘Y’ and ‘Z’ (Fig. 3). These rearrangements are visible only in this virus, alongside a low average protein similarity of ~ 50%, and may indicate that a fifth nudivirus genus could be erected to hold peracarid-infecting nudiviruses. We suggest *Epsilonnudivirus*. In further work, greater genomic availability of viruses from peracarid hosts could help to better define these demarcation criteria.

Further genomic characterisation of peracarid-infecting nudiviruses may also help to identify the evolutionary history of DhNV, especially with regards to genes that show relatedness outside the *Nudiviridae*. Examples include DhNV_029, which shares 35.19% similarity to the *cg30-1* gene (YP_009186763) from *Sucra jujuba nucleopolyhedrovirus* (Table 1), a butterfly-infecting baculovirus. This is the first of two ORFs with zinc finger, RING-type domains in the DhNV genome; with DhNV_045 being the second (Table 1). These do show some relation to HgNV and PmNV, where both HgNV and PmNV contain three proteins with Zinc finger, RING-type domains: HgNV_019, 064, and 067 and KN57gp_003, 033, and 049 respectively ^1,2^. DhNV_070 also shows high similarity to a non-nudivirus organism. A hypothetical protein from *Pyricularia oryzae* (Table 1), the fungal pathogen that causes rice blast disease, shows 41.18% similarity to DhNV_070. Protein domain analysis using InterProScan revealed mainly cytoplasmic and disorder protein domains from the *P. oryzae* sequence (XP_003712544) while DhNV_070 yielded a detailed signature match to proline rich extensin, commonly found in plant cell walls. This extensin domain does not appear in any *Gammanudivirus* protein. Finally, DhNV_076 shows some similarity to a homologue of HgNV (LOC108666550-like protein); however, both also show high levels of similarity to ORFs of invertebrate taxa, which lacks an identified domain or function. Such a conserved gene encoded by these viruses may reflect an ancient horizontal gene acquisition from a host during their evolutionary history.

### New perspectives surrounding the *Nudiviridae*

Nudivirus infections often delay development of their arthropod hosts, eventually causing death ^4^. However, high prevalence of nudiviruses in hosts apparently displaying few clinical signs of infection may also suggest some host benefit of retaining such sub-clinical infections ^2,5,10,11^. While the exact relationship between DhNV and host survival still requires testing, a significant association with increased activity may subsequently increase invasive capabilities of the host ^11^. Examining the genome of DhNV revealed several conserved and convergent traits of crustacean nudiviruses, highlighting potential genes for diagnostic development and further research into functional roles during host infection and survival within the environment. Further sequencing and characterisation of many hypothetical proteins will provide more insight into the evolutionary history and host relationship of DhNV relative to other *Nudiviridae*. Through genomic analysis, phylogeny, and virion morphology it is evident that the *Nudiviridae* in Crustacea are highly derived from their insect relatives and a great diversity of currently undescribed taxa likely reside in other arthropod hosts on land and in water.

## Materials and methods

### Collection of infected *Dikerogammarus haemobaphes* and next generation sequencing

*Dikerogammarus haemobaphes* were collected, dissected and underwent DNA extraction as explained by Bojko et al. ^11^, who also explore virion morphology and pathology associated with the discovery of a novel nudivirus. Stored DNA from a single individual displaying the characteristic pathology of bacilliform virus infection was selected for next generation sequencing using Illumina MiSeq and Illumina HiSeq. The DNA extract underwent library preparation for Illumina MiSeq using the NEXTERA XT library preparation kit, according to manufacturer’s protocol (Illumina, UK). The library underwent quality screening using a bioanalyzer (Agilent), was quantified using a QuantiFluor fluorimeter (Promega), was denatured using sodium hydroxide and diluted to 10 pM in Illumina HT1 hybridisation buffer for sequencing via an Illumina V3-600 cartridge. The same DNA extract was used to produce a library for Illumina HiSeq using the Illumina TruSeq DNA PCR-Free library preparation kit, according to manufacturer’s protocols. The library underwent quality screening using a bioanalyzer (Agilent), was quantified using a QuantiFluor fluorimeter (Promega), was denatured using sodium hydroxide and diluted to 10 pM in Illumina HT1 hybridisation buffer for sequencing on an Illumina HiSeq 3,000 with a 2 × 150 cartridge.

MiSeq and HiSeq outputs were trimmed in silico using Illuminaclip v0.32 (Trimmomatic: LEADING:3 TRAILING:3 SLIDINGWINDOW:4:15 MINLEN:36) ^14^ and pooled into paired and unpaired sequence files. The paired sequence data from each technique were paired-end-combined using PEAR v0.9.8 (settings: overlap similarity minimum, 20 bp) ^15^ to increase the read length of paired reads by combining the reads into single sequence reads. These reads were assembled using SPAdes v3.13.0 ^16^ with default parameters and k-mer lengths 21, 33, 55, 77, 99 and 127, to produce 228,433 scaffolds with a maximum read length of 119,824 bp and minimum read length of 128 bp.

### Identification and annotation of the viral genome

Scaffolds above 100,000 bp were extracted from the dataset and annotated using PROKKA v1.11 ^17^. The subsequent output was assessed for similarity to existing sequence data using NCBI, Blastp nr database. This identified a raw contiguous sequence of 119,824 bp as the genome of DhNV, which was subsequently circularized and checked for average coverage using CLC Genomics workbench v11 to result in a genome of 119,754 bp (coverage: 157.93X). PROKKA v1.11 ^17^ and GeneMarkS ^18^ was used to annotate the viral genome (parameters: virus). A combination of these two tools resulted in 95 identical open reading frames (ORFs), 8 frames with high similarity but different gene size and three ORFs identified just by PROKKA. Combined, this provided 106 ORFs for annotation. The protein product of the 106 ORFs were compared to existing information using BLASTp via the NCBI repository (GenBank) with a cut-off e-value of < 0.001. The protein sequences were also assessed using the InterProScan tool (ebi.ac.uk/interpro/) to identify domains and predicted function. Twenty-one conserved core baculovirus/nudivirus genes were identified; however, P6.9 was not found within the genome of DhNV after analysis using BLASTp, ExPASy ^19^, GeneMarkS and InterProScan.

The gene synteny data for DhNV was compared to two crustacean-infecting viruses, *Homarus gammarus nudivirus* (HgNV) and *Penaeus monodon nudivirus* (PmNV) (*Gammanudiviridae*), and an insect-infecting virus, *Tipula oleracea nudivirus* (ToNV) (*Deltanudiviridae*), whose data were obtained from NCBI accessions: KJ184318, MK439999, NC_026242, respectively. The data were plotted using Circa (omgenomics.com/circa).

The annotated viral genome is available through NCBI accession: MT488302.

### Phylogenetic analysis of DhNV among the *Nudiviridae*

A concatenated phylogenetic tree was developed using 18 of the 21 identified nudivirus core proteins from DhNV and seven other nudiviruses: *Drosophila innubila nudivirus* (DiNV), *Oryctes rhinocerous nudivirus* (OrNV), *Gryllus bimaculatus nudivirus* (GbNV), *Helicoverpa* (syn. *Heliothis*) *zea nudivirus*-2 (HzNV2), HgNV, PmNV, and ToNV. A baculovirus outgroup, *Lonomia obliqua multiple nucleopolyhedrovirus* (LoobMNPV) was used to root the tree. The p47 (missing from GbNV), Helicase 2, vlf-1, and p6.9 were not included as they are not present in the genomes of all the tested nudiviruses or are duplicated in DhNV. The remaining conserved proteins, *38 k*, *ac81, DNA polymerase, helicase, lef-4, lef-5, lef-8, lef-9, ac92 (p33), p74 (pif-0), pif-1, pif-2, pif-3, odv-e28* (*pif-4*)*, odv-e56 (pif-5), pif-6, vp91 (pif-8)* and *31 K (vp39),* were aligned using Geneious v10 using MAFFT with default parameters. In HzNV2, Lef-9 was trimmed using Geneious due to its fusion with p47 ^1^. IQtree was used to produce the maximum likelihood phylogenetic tree, which included 13,795 positions using the VT + F + I + G4 model (according to BIC) with 1,000 bootstrap replicates. Subsequently, the tree was imported into Figtree v1.4.3 for annotation. Transmission electron micrographs from each nudivirus were used to create illustrations of the virions with approximations of nucleocapsid size ^2,11,20–25^. A second concatenated tree was produced using putative *iap* and *pif-2* genes from DhNV and seven other nudiviruses: OrNV, GbNV, HzNV2, HgNV, PmNV, ToNV, in addition to recently obtained *Macrobrachium rosenbergii nudivirus* CN-SL2011 (MrNV) (NCBI:txid1217568) sequences, which includes just two protein coding genes (*iap* and *pif-2*). DiNV was excluded from this tree as it lacks an identifiable *iap* ORF. The baculovirus, *Lonomia obliqua multiple nucleopolyhedrovirus* (LoobMNPV) was used to root the tree. Genes were trimmed using Geneious v10 and aligned using MAFFT with default parameters. IQtree produced a phylogenetic tree using the Blosum62 + G4 model (according to BIC) with 1,000 bootstrap replicates. The tree was imported into Figtree v1.4.4 for final annotation.

**Table 1:**
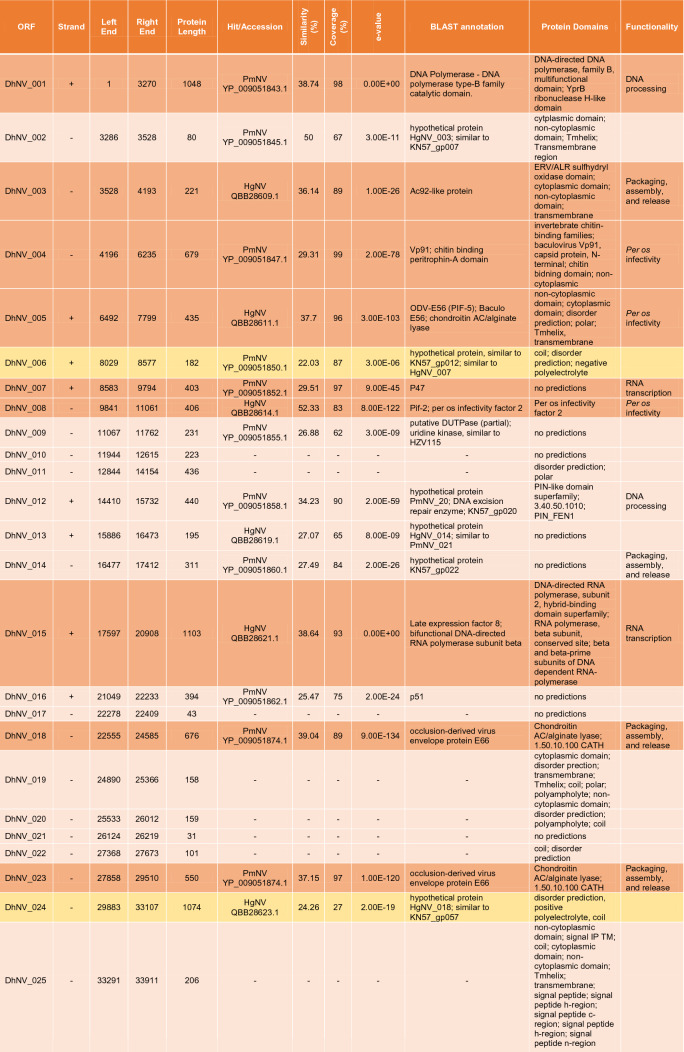

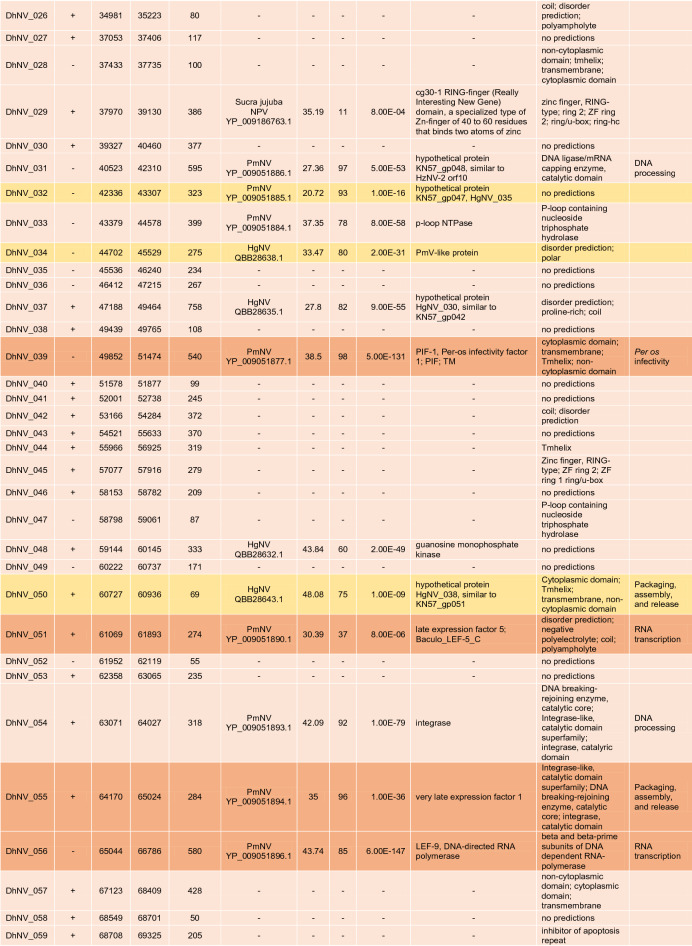

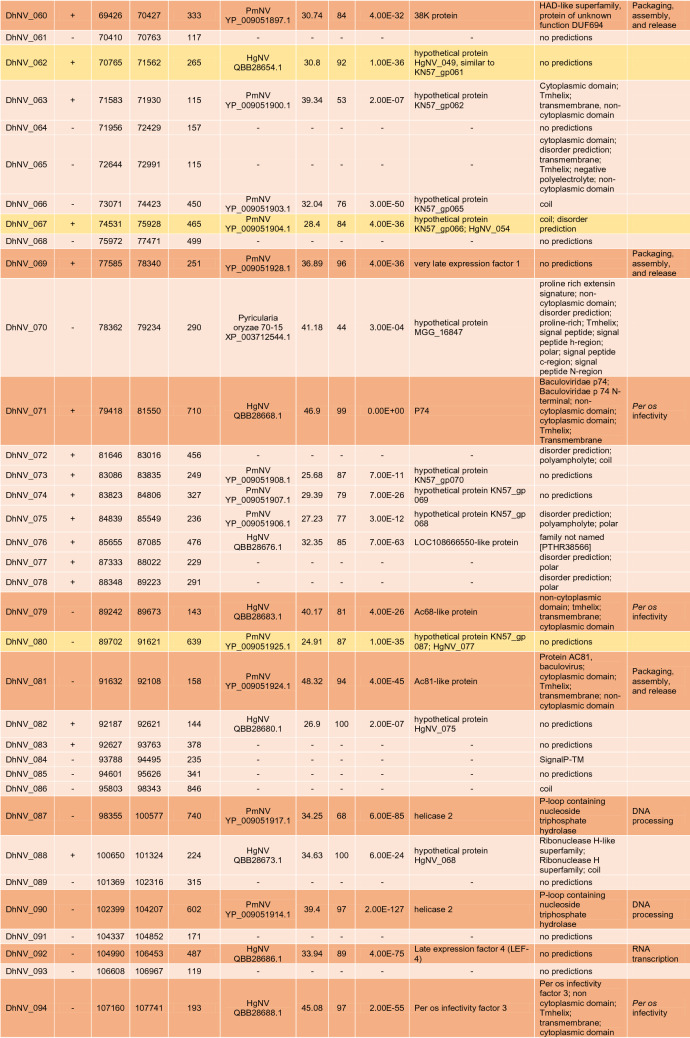

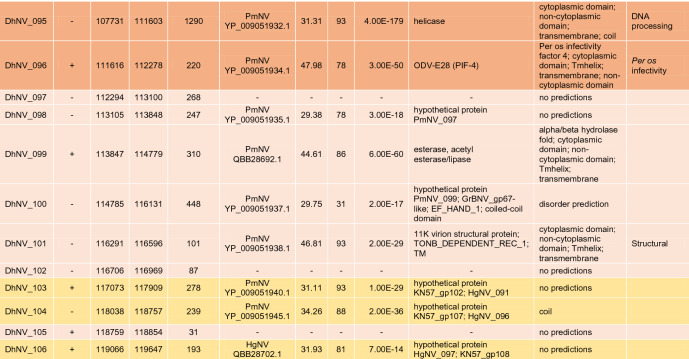
Table displaying identified ORFs of DhNV. Positive/negative strand, left end, right end, and protein length are listed.

Hit/Accession come from the top BLASTp result with an e-value < 0.001. Similarity, coverage, and exact e-values are displayed from said results. BLAST repository notes from each similar protein are shown along with identified protein domains and commonly identified nudivirus functional groups. Core genes (n = 24) are presented with a darker orange background and genes with similarity and function only detected in the crustacean viruses (n = 11) are noted in yellow

## Data Availability

Sequence data from this study are available through NCBI as stated herein. Biological materials from the host are available from the Cefas Aquatic Registry and Repository.
